# Effects of α2A Adrenoceptors on Norepinephrine Secretion from the Locus Coeruleus during Chronic Stress-Induced Depression

**DOI:** 10.3389/fnins.2017.00243

**Published:** 2017-05-01

**Authors:** Bin Wang, Ying Wang, Qiong Wu, Hong-ping Huang, Shao Li

**Affiliations:** ^1^Liaoning Provincial Key Laboratory of Cerebral Diseases, Department of Physiology, Dalian Medical UniversityDalian, China; ^2^Department of Physiology and Neurobiology, Wannan Medical CollegeWuhu, China; ^3^Department of Cardiology, Institute of Heart and Vessel Diseases of Dalian Medical University, The Second Affiliated Hospital of Dalian Medical UniversityDalian, China

**Keywords:** depression, locus coeruleus, hypothalamus, α2A-ardrenergic receptor, norepinephrine

## Abstract

Chronic stressors can often lead to the development of psychological disorders, such as depression and anxiety. The locus coeruleus (LC) is a stress sensitive brain region located in the pons, with noradrenergic neurons that project to the hypothalamus, especially the paraventricular nucleus (PVN) of the hypothalamus. The purpose of this paper is to better understand how alpha 2A-adrenoceptors (α_2A_-ARs) and LC-hypothalamus noradrenergic system participate in the pathophysiological mechanism of depression. *In vivo* norepinephrine (NE) release in the PVN triggered by electrical stimulation in the LC was detected with carbon fiber electrodes in depression model of rats induced by chronic unpredictable mild stress (CUMS). Also, the extracellular level of NE in the PVN was measured by microdialysis *in vivo* without any stimulation in the LC. The alpha 2-adrenoceptor (α_2_-AR) antagonist yohimbine and α_2A_-ARs antagonist BRL-44408 maleate were systemically administered to rats to determine the effects of α_2A_-ARs on NE release in the PVN. The peak value of elicited NE release signals in the PVN induced by electrical stimulation in the LC in the CUMS rats were lower than that in the control rats. The extracellular levels of NE in the PVN of the CUMS rats were significantly less than that of the control rats. Intraperitoneal injection of yohimbine or BRL-44408 maleate significantly potentiated NE release in the PVN of the CUMS rats. The CUMS significantly increased protein expression levels of α_2A_-AR in the hypothalamus, and BRL-44408 maleate significantly reversed the increase of α_2A_-AR protein expression levels in the CUMS rats. Our results suggest that the CUMS could significantly facilitate the effect of α2-adrenoceptor-mediated presynaptic inhibition and decrease the release of NE in the PVN from LC. Blockade of the inhibitory action of excessive α2A-adrenergic receptors in the CUMS rats could increase the level of NE in the PVN, which is effective in the treatment of depressive disorders.

## Introduction

Depression, a widespread mental disorder, influences over 10% of the world's population with profound social and economic consequences at any given time (Ferrari et al., 2014). Even though stress and monoamine neurotransmitter deficiency have been studied as two major causes of depression (Andrus et al., 2012) and researchers over the last 50 years have provided considerable evidence that the dysfunction of monoamine neurons is an important underlying pathology in major depressive disorder (Hamon and Blier, 2013), the detailed mechanisms related to its pathogenesis are still elusive. As a result, a number of patients fail to recover from chronic depression even though lots of medications have been applied to clinical treatment for depressive disorders, including NE reuptake inhibitors (NRIs), selective serotonin reuptake inhibitors (SSRIs), monoamine oxidase inhibitors (MAOIs), tricyclic antidepressants (TCAs), and antidepressants on DNA methylation patterns, etc. (Baudry et al., 2010; Massart et al., 2012; Kato and Chang, 2013).

Stress response is a risk factor that can develop anxiety, depression, posttraumatic stress disorder, and other affective or mental disorders, which is characterized by the activation of the locus coeruleus-norepinephrine (LC-NE) system (Altman et al., 1999; Ding et al., 2014). Stress can trigger the firing activity of noradrenergic neurons in the LC and subsequently widespread of NE transmission in the hypothalamus, prefrontal cortex, brainstem, cerebellum and amygdala (Liddell et al., 2005). The dysregulation of the paraventricular nucleus (PVN) of the hypothalamus contributes to behavioral and physiological alterations caused by chronic stress, and NE plays a prominent role in the PVN activation (Herman et al., 2008; Flak et al., 2014). Adrenergic receptors are located widely in the central nervous system and can be activated by NE. Three known alpha 2-adrenoceptor (α2-AR) subtypes, α_2A_-AR, α_2B_-AR and α_2C_-AR, are distributed in mammalian brain tissues (Bylund et al., 1994; Alexander et al., 2015), of which α_2A_-AR was identified as the predominant inhibitory autoreceptor in adrenergic neurons (Trendelenburg et al., 1993). Existing studies also demonstrate that both α_2A_-AR and α_2C_-AR subtypes play a role as presynaptic inhibitory receptors regulating neurotransmitter release, and α_2A_-AR subtype contributes more to presynaptic negative feedback inhibition of NE release in mice (Altman et al., 1999; Bücheler et al., 2002; Gyires et al., 2009). Lacking the α_2A_-AR in mice, presynaptic autoinhibition mediated by endogenous NE or α2-receptor agonists was significantly blunted but not absent (Altman et al., 1999).

We hypothesized that LC noradrenergic neurons projecting to the hypothalamus (PVN) may functionally participate in the pathogenesis of depression and the α_2A_-AR plays a principal role by modulating NE release. To confirm the hypothesis, we measured the NE release signal in the PVN evoked by electrical stimulation in LC through amperometric detection with carbon fiber electrode. We also detected the extracellular level of NE in the PVN by microdialysis *in vivo*. Western blot analysis was carried out to evaluate the expression level of α_2A_-AR in the hypothalamus.

## Materials and methods

### Animals

Male Wistar rats weighing 180–200 g (purchased from Animal center of Nanjing Qinglongshan and the Animal Experimental Center of Dalian Medical University) were used in our research. Animals were housed at the conventional dwelling unit under standard conditions (5 per cage, room temperature of 24°C, relative humidity of 45–65%, 12 h light/dark cycle), *ad libitum*. All experiments were carried out under the guidelines of the National Institutes of Health Guide for the Care and Use of Laboratory Animals.

### Chronic unpredictable mild stress model

Rats were divided into chronic unpredictable mild stresses (CUMS: rats exposed chronically to a variety of mild unpredictable stressors for 4 weeks) group (*n* = 20) and control group (*n* = 20) randomly (Willner et al., 1987). Each rat which belonged to the CUMS group was housed in one cage and subjected to one stressor one time a day (stressors included: water deprivation (15-h), cage tilt at a 45 degree angle (2-h), housing in mild damp sawdust (20-h), horizontal vibration (5-min), food deprivation (15-h), forced swim in water at 21°C (30-min) and intermittent white noise (85 dB, 3-h). All stressors lasted for 4-w and were applied at different points of time every week to avoid habituation and to provide an unanticipated feature to the stressors as described in detail previously (Shao et al., 2010; Wang et al., 2015). The control rats were housed in bigger cages (5 rats per cage) and they remained socially active.

### Behavioral tests

Behavioral tests included sucrose consumption test and open-field test. Sucrose consumption test was carried out as follows: two bottles of 1% sucrose water were randomly located in every cage at the first 2 days, which were turned into two bottles of tap water at the third day. Following with 15-h deprivation of food and water intake, a bottle of tap water and a bottle of 1% sucrose water were given to the rats. The consumption amount of 1% sucrose and total water were measured in the next 2-h. The sucrose preference percentage was calculated according to the following formula: Sucrose preference = sucrose intake (g)/[(sucrose intake (g) +water intake (g)] (Cui et al., 2014).

Open-field test was carried out to all the rats. Each rat was placed in the center of a white square box (length, 55 cm; width, 39 cm; height, 20 cm) for a 5-min observation. During the 5-min observation, horizontal and vertical exploratory locomotor activities were scored for the test.

### Amperometric detection of NE signals with carbon fiber electrode

Amperometric detection of NE signals with carbon fiber electrode was performed according to our previously described method (Gong et al., 2015). Rats were anesthetized with pentobarbital (50 mg/kg, i.p.), and fixed at the stereotaxic instrument (Life Technology Co. Ltd. of Shenzhen City). A bipolar stainless steel electrode (diameter: 1.0 mm) sent electrical stimulation (Isolated Pulse Stimulator model 2100; A-M Systems) into LC (A: − 10.0 mm; L: ± 1.4 mm; V: − 7.5 mm) according to the rat brain atlas (Paxinos and Watson, 1986). The amperometry working electrode was a cylindrical carbon-fiber electrode insulated by a glass capillary. The detecting carbon fiber electrode was inserted into the PVN (A: − 1.5 mm; L: ± 0.4 mm; V: − 8.5 mm). The reference electrode was a silver wire coated with AgCl and connected to the neck muscle tissue. A patch-clamp amplifier (PC-2B, INBIO, Wuhan, China) was applied under voltage-clamp mode, with the gain of 0.5 mV/pA and a CFE voltage of a constant + 700 mV for amperometry. All data were low pass filtered at 20 Hz and acquired by a data acquisition system with a digital interface and software (iPDA-0.1; INBIO, Wuhan, China). Norepinephrine release signals evoked by electrical stimulation (1.0 mA, 100 Hz, 100 pulses) in LC *in vivo* were analyzed. After recording stable NE signal, yohimbine (Sigma–Aldrich, 3 mg/kg, intraperitoneal injection) (Paalzow and Paalzow, 1983; McAllister, 2001) was administered to the rat and NE release signal was recorded again 30 min later to assess the function of α_2_-AR. The rats were euthanized with isoflurane and the whole brains were fixed in 10% formalin solution to verify the brain region.

### *In vivo* microdialysis

*In vivo* microdialysis was carried out according to a previously described method (Niwa et al., 2007). Rats were anesthetized with pentobarbital (50 mg/kg, i.p.) and were fixed in a stereotactic instrument (Life Technology Co. Ltd. of Shenzhen City). The intracerebral guide cannula (MBR-10, BASI, West Lafayette, IN 47906, USA) was implanted 1 mm above the PVN (A: − 1.5 mm, L: ± 0.4 mm; V: − 8.5 mm) and was secured onto the skull by stainless screws and dental acrylic cement. After 24 h, a microdialysis probe (MBR-1-10, 1 mm membrane length, BASI) was embedded into the guide cannula, and the ACSF was continuously perfused into the PVN through the probe. During the microdialysis experiments, dialysates were collected in 1-h increments at a velocity of 1 μL/min, and then 50 μL aliquots were used to measure NE levels with an ELISA kit (CSB-E07022r, CUSABIO, Wuhan, China). BRL-44408 maleate (Sigma-Aldrich, 3 mg/kg, i.p.) was administered to rats and NE dialysates were collected again to assess the function of α_2A_-AR (Miksa et al., 2009).

### Western blot studies

The protein from the hypothalamus (the hypothalamic tissue was dissected as the center between gray nodules and optic chiasma prechiasmal border as prozone, back of corpus albicans as posterior and bitemporal groove on both sides; about 4 mm width, 2 mm depth and 4 mm length) was extracted by using an extraction kit (Keygen Biotech, China), and the protein content was measured by a BCA protein assay (Keygen Biotech, China). For Western Blotting, the proteins (20 μg) for each sample were loaded into a 10% SDS-polyacrylamide gel for electrophoresis. Then, the protein components were transferred to polyvinylidene difluoride (PVDF) membranes, and then blocked with 5% BSA in TBST (TBS+0.1% Tween-20) for 1 h, and then immunoblotted overnight at 4°C with primary antibody for α_2A_-AR (#14266-1-AP, Proteintech, USA). Subsequently, membranes were washed three times in TBST and incubated with a horseradish peroxidase-conjugated secondary antibody (anti-rabbit, 1:5,000, ZSJQ-BIO Company, Beijing, China) for 1 h at room temperature. The infrared band signals were detected using BIO-RAD (Hercules, CA, USA) gel analysis software. The blots were then washed with TBST, blocked for 1 h and incubated with the primary antibody β-actin (ab6276, Abcam), for loading control. The Densitometric analysis of immunoreactivity was conducted using the NIH Image J software and normalized to the immunoreactivity of the control rats.

### Statistical analyses

The data were analyzed using GraphPad Prism (GraphPad Software Inc.) and SPSS 21.0, expressed as mean ± SEM., and statistical analyses were performed using a paired *t*-test or an unpaired Student's *t*-test for two-sample comparison, two-way ANOVA was used to evaluate the effects of antagonists between groups (**Figures 2C**, **3B**, **4B**), and the microdialysis data summarized in Figure **3A** were assessed by repeated-measures ANOVA. Significance was set at p < 0.05.

## Results

### Rat-specific depressive behavior induced by CUMS

After 4 weeks of CUMS, rats in the model showed a significant reduction in body weight compared to that in the control rats (*p* < 0.01, *n* = 20, respectively). Sucrose preference is frequently used as a measure of anhedonia in rodents (Gilsbach et al., 2009). Significant reductions of sucrose intake (*p* < 0.01) and sucrose preference (*p* < 0.05) were detected in the CUMS rats. The rats of CUMS group showed a significant reduction in the horizontal (*p* < 0.01) and vertical (*p* < 0.05) exploratory locomotor activity (Table 1).

**Table 1:** **The body weight, open-field test and sucrose consumption test in the two groups rats after modeling (***n*** = 20, mean ± SEM)**.

	**Body weight (g)**	**Open field test**			**Sucrose consumption**		
		**Horizontal score**	**Vertical score**	**Total score**	**Sucrose (g)**	**Total (g)**	**Sucrose preference (%)**
Control	306.34 ± 7.2	37.3 ± 3.96	14.5 ± 2.03	51.8 ± 5.47	9.2 ± 0.87	12.74 ± 0.99	71.9 ± 2.6
CUMS	268.86 ± 4.88^**^	16.9 ± 1.35^**^	7.9 ± 1.32^*^	24.8 ± 1.50^**^	5.6 ± 0.40^**^	8.76 ± 0.68^**^	64.1 ± 2.2^*^

CUMS produced a significant decrease in the body weight, horizontal and vertical exploratory locomotor activity, sucrose intake and sucrose preference in the CUMS rats compared to that in the control rats (

* *p < 0.05*,

** *p < 0.01)*.

In our experiment, the CUMS rats showed significantly lower body weight, less locomotor activity in the open field test and lower sucrose preference ratio in the sucrose consumption test than that of the control rats after CUMS, which meant the CUMS induced depression successfully.

### The LC-PVN noradrenergic system participated in the depression induced by CUMS

**Figure 1:**
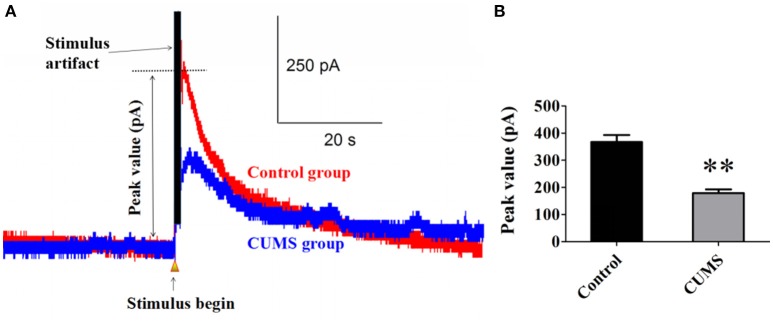
**CUMS significantly decreased the peak value of elicited NE release signal (A)** Representative raw data of typical NE release signals recorded in the control and CUMS group rats. The peak value was labeled in figure. **(B)** The peak value of NE signal in the PVN was significantly decreased in the CUMS rats. ^**^p < 0.01 vs. the control group rats.

Norepinephrine is the main neurotransmitter in noradrenergic system. Elicited NE release from noradrenergic nerve fibers in the PVN induced by electrical stimulation in LC was detected with carbon fiber electrode. The data showed that chronic stresses significantly decreased the peak value of elicited NE release signal. There were statistical differences between the CUMS group and control group (179.1 ± 13.5 pA vs. 367.1 ± 26.2 pA, *n* = 9, *p* < 0.01. Figures 1A,B). This result demonstrated that the LC-PVN noradrenergic system participated in the CUMS-induced depression.

### The α2-AR participated in pathophysiology of depression induced by the CUMS

**Figure 2:**
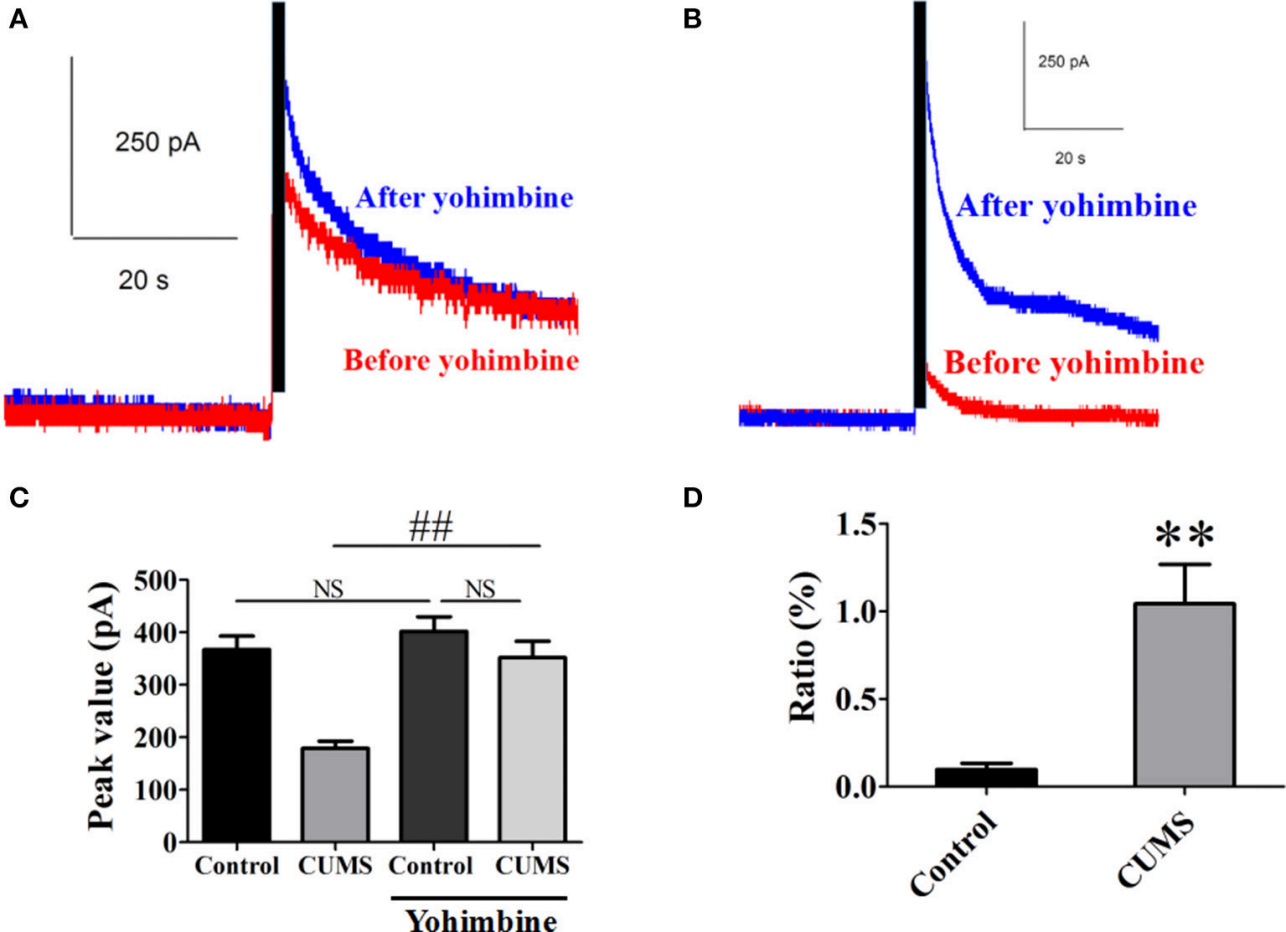
**Intraperitoneal injection of yohimbine potentiated elicited NE release signal in the CUMS rats. (A)** Representative raw data of typical NE release signals recorded in the control rats before and after yohimbine. **(B)** Representative raw data of typical NE release signals recorded in the CUMS rats before and after yohimbine. **(C)** Yohimbine significantly increased the peak value of NE in the PVN of the CUMS rats, but no significant difference was observed in the control rats. **(D)** The ratio of increase in the peak value of NE signal was significantly amplified after administration of yohimbine in the CUMS rats compared to that in the control rats. The ratio: [(after-before)/before yohimbine]. ^**^p < 0.01 vs. the control rats, ^*##*^*p* < 0.01 vs. before yohimbine administration in the CUMS group. NS represents no significance.

Yohimbine is one of the α_2_-AR antagonists. Intraperitoneal administration of yohimbine (3 mg/kg, i.p.), potentiated the peak value of NE release signal in the PVN of each group of rats evoked by electrical stimulation in LC [for CUMS group, 351.9 ± 31.2 pA vs. 179.1 ± 13.5 pA, *n* = 9, *p* < 0.01; for control group, 401.8 ± 28.2 pA vs. 367.1 ± 26.2 pA, *n* = 9, *p* > 0.05. *F*_(3, 32)_ = 15.76. Figures 2A–C]. The ratio of increase in the peak value of NE release signal in the CUMS rats was significantly amplified after the yohimbine administration compared to that in the control rats (104.3 ± 22.5% vs. 99% ± 3.5%, *n* = 9, *p* < 0.01. Figure 2D). These results confirmed that α_2_-AR acted to inhibit NE release and participated in the pathophysiology of depression induced by the CUMS.

### Effects of BRL-44408 maleate on the extracellular level of NE in the PVN

**Figure 3:**
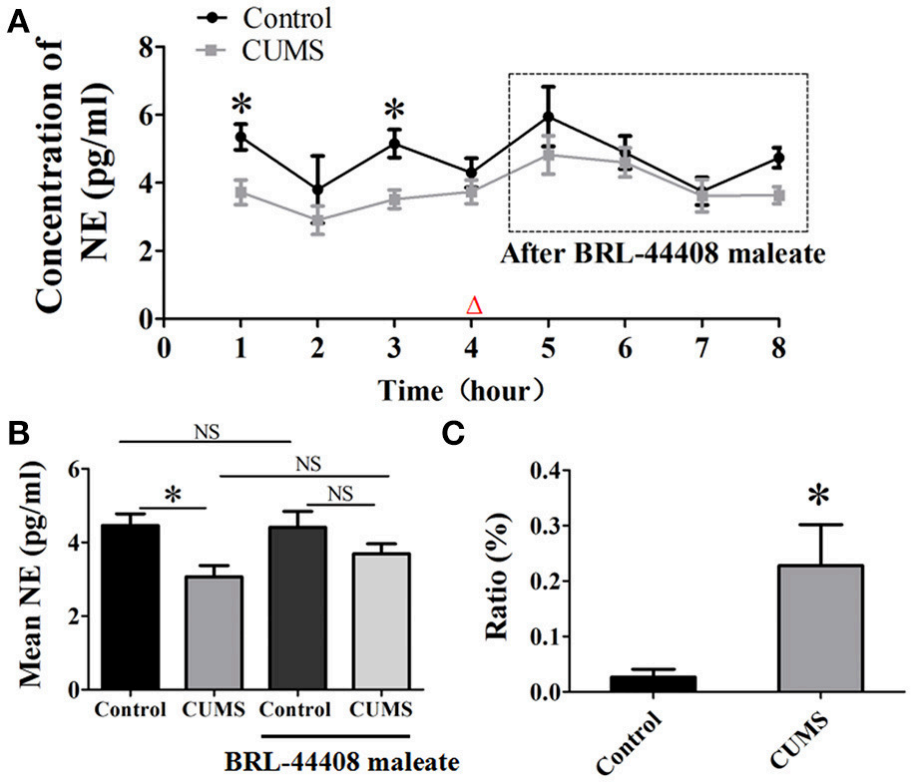
**Intraperitoneal injection of BRL-44408 maleate can reverse the decrease of NE release induced by CUMS. (A)** The dialysate concentration of NE were less in the first and third hour in the CUMS rats than that in the control rats, intraperitoneal injection of BRL-44408 maleate increased extracellular level of NE in both group of rats, but no significance was observed between the two groups. **(B)** The CUMS significantly decreased the mean levels of extracellular NE in the PVN of the CUMS rats compared to that in the control rats, but no significance was observed between the two groups after BRL-44408 maleate. **(C)** The ratio of increase in the NE release was significantly amplified after administration of BRL-44408 maleate in the CUMS rats compared to that in the control rats. The ratio: [(after-before)/before BRL-44408 maleate]. Δ: BRL-44408 maleate administration (3 mg/kg, i.p.). ^*^p < 0.05 vs. the control rats. NS represents no significance.

The dialysate concentration of NE was significantly decreased in the first (3.7 ± 0.4 vs. 5.3 ± 0.4, *p* < 0.05, *n* = 5) and the third hour (3.5 ± 0.3 vs. 5.2 ± 0.4, *p* < 0.05, *n* = 5) in the CUMS rats compared to that in the control rats, no significant difference was observed in the second and forth hour (Figure 3A). Repeated-measures ANOVA demonstrated a significant group × time interaction for NE [*F*_(1.995, 7.982)_ = 4.996, *p* = 0.039], but no significance within-subjects effects (for time). Mean concentrations of NE in the PVN of the CUMS rats were less than that of the control rats [3.1 ± 0.3 vs. 4.3 ± 0.4, *p* < 0.05, *n* = 5. *F*_(3, 16)_ = 5.973. Figure 3B].

The alpha 2A-adrenoceptors antagonist BRL-44408 maleate significantly amplified the ratio of increase [(after-before)/before BRL-44408 maleate] of NE in the CUMS rats compared to that of the control rats (22.8 ± 7.4% vs. 2.7 ± 1.4%, *n* = 5, *p* < 0.05. Figure 3C). These results suggest the inhibitory action of α_2_-AR on LC-PVN noradrenergic system maybe partly through α_2A_-AR subtype in the CUMS rats.

### Western blot studies

**Figure 4:**
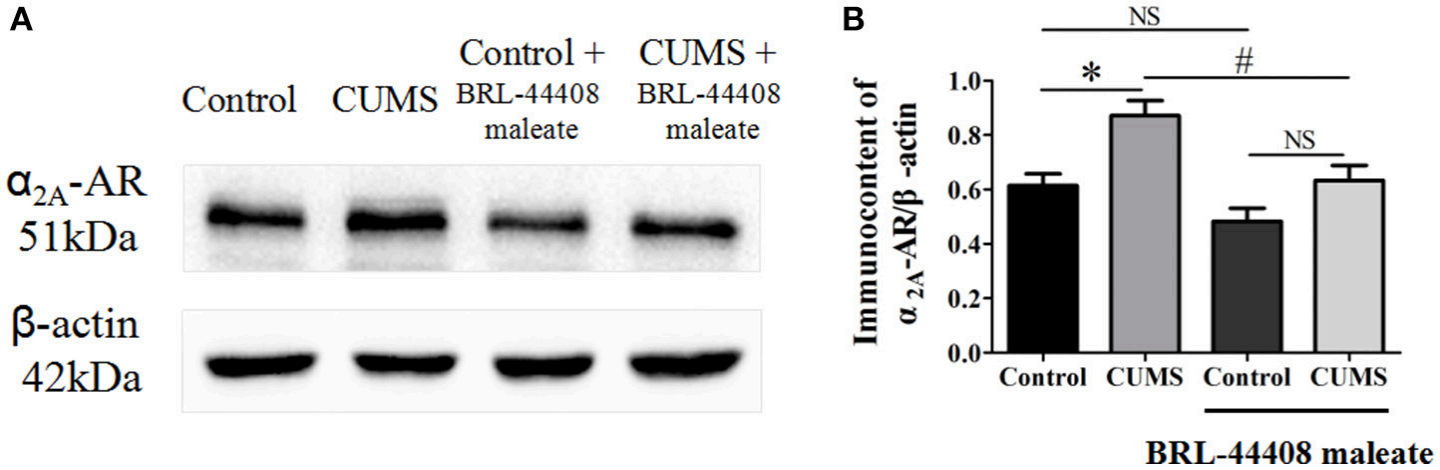
**Intraperitoneal injection of BRL-44408 maleate can reverse the increase of α_**2A**_-AR induced by CUMS in the hypothalamus of the CUMS rats**. **(A.B**) The protein expression levels of α_2A_-AR was significantly increased in the hypothalamus of the CUMS rats, peripheral administration of BRL-44408 maleate significantly decreased the expression levels of α_2A_-AR in the CUMS rats. ^*^p < 0.05 vs. the control rats. ^#^*p* < 0.05 vs. before BRL-44408 maleate in the CUMS group. NS represents no significance.

The protein expression levels of α_2A_-AR (observed at 51 kDa) was significantly increased in the hypothalamus of the CUMS rats compared to that of the control rats (0.87 ± 0.05 vs. 0.61 ± 0.04, *p* < 0.05, *n* = 5). BRL-44408 maleate significantly decreased the α_2A_-AR protein level in the CUMS rats (0.63 ± 0.05 vs. 0.87 ± 0.05, *p* < 0.05, *n* = 5), but no significance was observed in the control rats (0.48 ± 0.05 vs. 0.61 ± 0.04, *p* > 0.05, *n* = 5). [*F*_(3, 16)_ = 12.18, Figure 4]. The results demonstrated that CUMS increased α_2A_-AR level in the hypothalamus and the increased quantity of α_2A_-AR contributes to decreased NE release in the hypothalamus.

## Discussion

The CUMS model was developed based upon the hypothesis of depression induced by stress. Antidepressants agents can reverse most effects of CUMS, illustrating a strong predictive validity of this model for depression. However, the mechanisms underlying the CUMS are still not understood completely. Our results showed that the CUMS significantly decreased the peak value of elicited NE release in the PVN evoked by electrical stimulation in LC, which illustrated that the secretion of NE from LC projecting to the PVN nerve fiber endings were decreased in the CUMS rats compared to that in the control rats.

Stressors can damage LC (Samuels and Szabadi, 2008). Moreover, damage or loss of LC noradrenergic neurons could result in the decrease of NE in the central nervous system (Marien et al., 2004; Rommelfanger and Weinshenker, 2007; Weinshenker, 2008). In our study, we found yohimbine, α_2_-AR antagonist (Makau et al., 2016), significantly increased the peak value of elicited NE release from noradrenergic nerve fibers in the PVN evoked by electrical stimulation in LC in the CUMS rats. It is generally recognized that the α2-adrenergic receptors are coupled with the inhibitory guanosine triphosphate (GTP)-binding protein, which may be involved in the receptor-mediated transmembrane signaling by regulating adenylate cyclase activity (Tsuda et al., 2003). This further weakens the calcium current mediated by voltage-gated calcium channels and the potassium current reliant on the calcium ions, then causes a decrease in the concentration of cytoplasm Ca^2+^, in turn inhibits the synthesis and release of NE (Abdulla and Smith, 1997). Yohimbine increases the release of NE in the PVN by inhibiting the signaling pathway of α_2_-AR, suggesting that the functional change of the presynaptic membrane α2 receptor has a connection with the targets for the treatment of depression.

We used microdialysis to estimate the extracellular level of NE in the PVN in order to confirm the decreased NE level in the PVN of the CUMS rats and eliminate the interference of 5-HT and dopamine in the amperometric detection of NE. The results showed that the CUMS significantly decreased the levels of NE in the PVN, and selective α_2A_-ARs antagonist BRL-44408 maleate significantly increased the levels of NE in the PVN of the CUMS rats compared to that in the control rats. Our data suggest that blockade of α2A-adrenergic receptor can increase the level of NE in the PVN. *In vivo* dialysate measured by microdialysis showed α2A-adrenergic receptor agonist clonidine decreased the level of NE in the prefrontal cortex (Doucet et al., 2013), LC and cingulate cortex (Mateo et al., 2001), which also indicate that NE release might be highly dependent on the α2A-adrenergic receptors. Hence, the anomaly of α_2A_-ARs maybe a physiopathology mechanism to trigger depressive disorder through direct or indirect effects to the secretion of NE in the PVN and the firing of LC noradrenergic neurons (Aoki et al., 1994; Nörenberg et al., 1997; Lee et al., 1998; Guiard et al., 2008; Wang et al., 2009).

The results showed that the expression levels of α_2A_-AR in the hypothalamus were significantly increased in the CUMS rats compared to that in the control rats, and the up-regulated effects of α_2A_-AR were significantly reversed by the acute administration of BRL-44408 maleate. These findings suggest that the CUMS up-regulates the quantity of α_2A_-AR and the α_2A_-ARs antagonist, BRL-44408 maleate, may block the activities of α_2A_-ARs. Post mortem studies in prefrontal cortex, hippocampus and LC of depressed patients revealing up-regulation of α_2A_-ARs, and elevated α_2A_-ARs RNA expression in the glutamatergic neurons induced by chronic stress (Flügge et al., 2003). However, α_2A_-AR knockout mice showed depressive-like behavior that was not responsive to imipramine, and therefore was concluded that α_2A_-AR has antidepressive effects (Schramm et al., 2001). This reminds us that the increase in α_2A_-AR expression in the CUMS rats might be regarded as a compensatory rebound effect, possibly because of decreased amounts of NE primarily at sites distant from the LC. Excessive expression of α_2A_-AR may suppress adrenaline-induced (cAMP)_i_ increase and exocytosis (Harada et al., 2015). Since α_2A_-ARs are generally coupled with Gαi/o proteins, the overexpression of α_2A_-AR can promote Gi function, resulting in the inhibition of cAMP production and a series of intracellular signal transmission, eventually leading to inhibition of neuron activity and NE secretion. In our study, the increased expression levels of α_2A_-AR in the CUMS rats may have induced the inhibition of the secretion of NE through activation of Gi protein signal pathway (Wu and Saggau, 1997; Brown and Sihra, 2008). The alpha 2A-adrenoceptors antagonist BRL-44408 maleate blocked the excessive α_2A_-AR and down-regulated the quantity of α_2A_-AR, which could weaken or eliminate its inhibitory effect to NE release.

However, although α_2A_-ARs contributes the significantly inhibitory effect on NE release, other subtypes of α_2_-AR, such as α_2B_-AR and α_2C_-AR may have less effect on NE release, indicating that α2-adrenoceptors antagonists might be better drugs for the treatment of depression. It also cannot be ignored that yohimbine also augmented anxiety both in human and rodents (Davis et al., 1979; Morgan et al., 1993; Altobelli et al., 2001), and the enhanced central noradrenergic activity is associated with the activation of fear and anxiety circuitries. This paradox may precisely result from the increase of NE by yohimbine, which may induce the activation of stimulatory α1- and of β-adrenergic receptor, the latter may mediate the enhancement of neuronal activity and further induce the activation of anxiety (Montoya et al., 2016). Hence, the dose and duration of α2-adrenoceptor antagonists for the treatment of depression require future research.

## Conclusion

Our results suggest that the CUMS could significantly facilitate the effect of α2-adrenoceptors-mediated presynaptic inhibition and decrease the release of NE in the PVN from LC. Blockade of the inhibitory action of the excessive α_2A_-adrenergic receptor in the CUMS rats could increase the level of NE in the PVN, which is effective in the treatment of depressive disorders.

## Ethics statement

This study was carried out in accordance with the recommendations of National Institute of Health Guide for the Care and Use of Laboratory Animals (NIH Publications No. 80-23) revised 1996. All experimental protocols were approved by the animal studies committees of the Dalian Medical University and the committee on the Ethics of Animal Experiments of the Wannan Medical College, and all efforts were made to minimize the number of animals used and their suffering.

## Author contributions

BW: Substantial contributions to design of the work; the acquisition of data for the work; writing of the article. YW: Assist the experiment to finish, the analysis and interpretation of data for the work. QW: Assist the experiment to finish, initial article revision. HH: Contributions to the conception of the work, ultima article revision. SL: Contributions to the conception of the work, final approval of the version to be published.

### Conflict of interest statement

The authors declare that the research was conducted in the absence of any commercial or financial relationships that could be construed as a potential conflict of interest.
